# Management of a Mass Casualty Incident Involving Foreign Workers: Insights From a Single-Center Response to a Microbus Accident in Japan

**DOI:** 10.7759/cureus.78084

**Published:** 2025-01-27

**Authors:** Rina Shibayama, Yujo Kawashita, Noriko Ikeda, Wakana Yasumoto, Kazuki Kanazawa, Masaki Tateishi, Mitsuhiro Yasuda, Toshiro Okuyama, Hiroyuki Ishida, Tsubasa Sakai, Sousei Abe, Junzo Yamaguchi, Yoshinobu Horio, Yukihiro Sugimoto, Mio Nakazato, Takashi Ueda

**Affiliations:** 1 Surgery, Fukuoka Seisyukai Hospital, Fukuoka, JPN; 2 Orthopaedics, Fukuoka Seisyukai Hospital, Fukuoka, JPN; 3 Emergency Medicine, Fukuoka Seisyukai Hospital, Fukuoka, JPN; 4 Plastic Surgery, Fukuoka Seisyukai Hospital, Fukuoka, JPN; 5 Surgery, Fukuoka Seisyukai Hospital, Fukuoka prefecture, JPN; 6 Neurosurgery, Fukuoka Seisyukai Hospital, Fukuoka, JPN; 7 Pulmonology, Fukuoka Seisyukai Hospital, Fukuoka, JPN; 8 General Practice, Fukuoka Seisyukai Hospital, Fukuoka, JPN

**Keywords:** acute trauma care, emergency medical service, emergency service, road traffic injuries, triage protocols

## Abstract

Background: The increasing globalization of workforces presents unique challenges to emergency medical services worldwide. Japan, traditionally a homogeneous society, now faces a growing need to manage multicultural emergency scenarios. This study examines a mass casualty incident (MCI) predominantly involving foreign workers, highlighting the challenges and strategies for effective emergency care amid language and cultural barriers.

Methodology: We conducted a comprehensive analysis of emergency response management for an MCI involving 20 patients (18 foreign nationals and two Japanese nationals) at a secondary emergency medical facility in Japan. Our protocol integrated a two-tier triage system (Simple Triage and Rapid Treatment, followed by Physiological and Anatomical Triage) with a multicultural communication strategy.

Results: Initial triage categorized 17 patients as green (minor) and three as yellow (delayed), with two patients requiring subsequent reclassification from green to yellow. Assessment times for foreign nationals averaged significantly longer than those for Japanese patients (22.3 minutes vs. 12.5 minutes). Implementation of digital translation tools and multilingual medical cards effectively bridged communication gaps. The majority of patients (15/17) were successfully treated and discharged, while two required specialist referral. No fatalities or severe complications were recorded.

Conclusions: This case study emphasizes the significance of three critical components for the effective management of multicultural MCIs. First, dynamic triage protocols must be designed to accommodate language barriers, ensuring equitable and timely care for all patients. Second, the integration of advanced technological solutions for real-time medical translation is essential to bridge communication gaps and support clinical decision-making during emergencies. Finally, culturally competent emergency care systems are vital for addressing the specific needs of diverse patient populations and fostering trust in healthcare delivery. As workforce globalization continues to advance, these findings provide practical insights for emergency medical services adapting to the challenges of increasingly diverse populations. These results have particular relevance for developed nations experiencing demographic transitions through immigration.

## Introduction

Japan's aging population has led to an increased reliance on foreign workers, presenting unique challenges in emergency medical care [1]. As of 2024, foreign workers constitute a growing segment of Japan's workforce, particularly in manufacturing and service industries. This demographic shift has created new demands on the emergency medical system, especially in regions with high concentrations of foreign workers [2]. Effective triage and management of mass casualty incidents (MCIs) involving foreign nationals have become increasingly critical due to language and cultural barriers [3].

This study aims to achieve the following objectives:

Evaluate the effectiveness of existing triage protocols in managing an MCI involving foreign nationals. Identify challenges posed by language and cultural barriers during the emergency response. Assess the role of technological tools, such as translation devices, in improving communication and care delivery. Propose actionable strategies for enhancing emergency response protocols for multicultural patient groups.

By addressing these objectives, the study seeks to provide practical insights for emergency medical services in an increasingly multicultural context.

## Materials and methods

Study design and setting

This observational study analyzes an MCI response at Fukuoka Seisyukai Hospital, a 213-bed secondary emergency medical facility in Fukuoka, Japan. The hospital serves as a regional emergency medical center and is located 1.8 km from the accident site. The study protocol was reviewed by the institutional review board, which waived the requirement for informed consent due to the retrospective observational nature of the study.

Patient population and initial response

The study encompassed all casualties from a single microbus accident that occurred on January 25, 2024. To maintain the comprehensive nature of the MCI analysis, all patients involved in the incident were included, regardless of injury severity or nationality. The initial emergency response was activated following established institutional protocols for MCIs.

**Figure 1:**
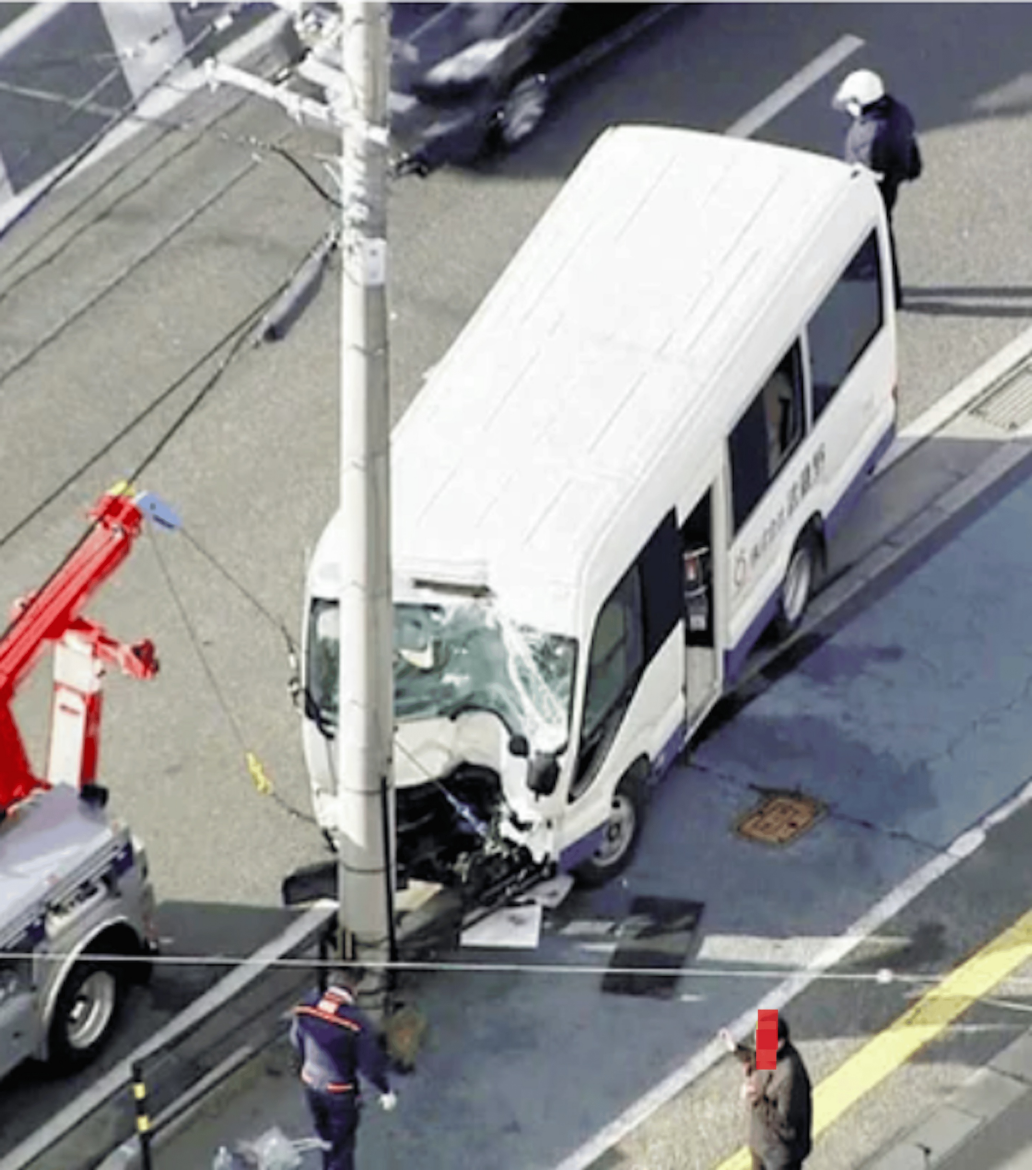
Overview of the mass casualty incident. High-speed impact (50-60 km/hour) collision between a microbus and a utility pole. Time: 6:30 AM, January 25, 2024 Location: Prefectural road, Kasuya town Patients: Total 20 (16 Nepalese, two Filipino, and two Japanese nationals) Image courtesy of NHK (Japan Broadcasting Corporation), reproduced with permission [4].

Figure 1 illustrates the scene of the incident, where a microbus carrying 20 passengers collided with a utility pole at a speed of 50-60 km/hour. The structural damage to the vehicle and the distribution of patients at the scene guided our initial emergency response strategy.

Triage and assessment protocols

Initial field triage was conducted using the Simple Triage and Rapid Treatment (START) method, which evaluates patients based on mobility, respiratory function, circulatory status, and consciousness level [5]. This protocol was selected for its established reliability in mass casualty scenarios and its widespread adoption in emergency medical services. Secondary triage employed the Physiological and Anatomical Triage (PAT) method, incorporating both physiological parameters and anatomical injury patterns to enable more detailed patient assessment [6].

**Figure 2:**
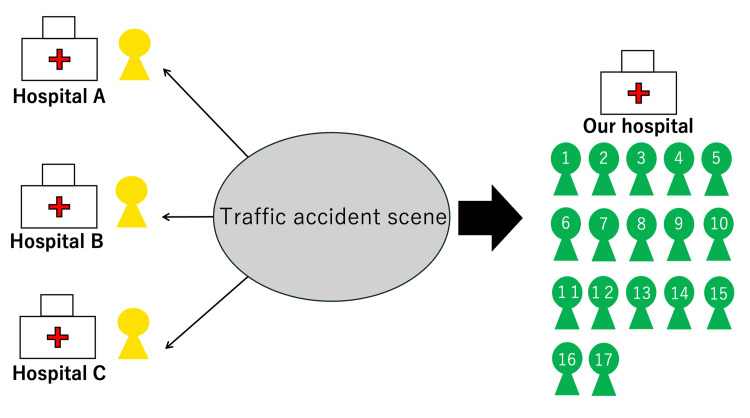
Scene triage and patient transport destinations. A schematic representation of the initial scene triage conducted using the Simple Triage and Rapid Treatment (START) method. Three patients classified as yellow (delayed) were transported to nearby hospitals (Hospitals A, B, and C), while the 17 green-tagged patients (minor injuries) were transferred to Fukuoka Seisyukai Hospital for further assessment and treatment.

The distribution of patients following initial triage is depicted in Figure 2, demonstrating the systematic approach to patient allocation between our facility and neighboring hospitals.

This allocation was based on both injury severity and facility capacity, ensuring optimal resource utilization across the regional emergency medical network.

Communication and language support system

A multi-modal communication strategy was implemented to address language barriers, comprising electronic translation devices equipped with specialized medical terminology databases and multilingual medical communication cards. Visual communication aids supplemented verbal communication when necessary. The hospital's electronic medical record system was configured to accommodate multiple language inputs while maintaining standardized medical documentation.

Data collection and analysis

A structured data collection protocol was implemented to capture comprehensive information about the incident response. Patient demographic data included age, gender, and nationality. Temporal metrics documented the progression from incident occurrence through various stages of medical care. Clinical parameters encompassed initial presentation, injury patterns, therapeutic interventions, and final disposition.

## Results

Patient characteristics and demographics

The study population comprised 20 individuals involved in the microbus accident, representing a diverse demographic profile. The majority were foreign nationals (*n *= 18), including 16 Nepalese and two Filipino citizens, with two Japanese nationals completing the cohort. Female patients predominated, accounting for 13 of the 20 casualties, with an age distribution ranging from 22 to 64 years (mean age: 34.7 years).

Triage outcomes and patient flow

The first wave of patients arrived at 7:35 AM, before the daytime shift change. The hospital staff at that time included five doctors, seven nurses, and five administrative staff. This team efficiently managed the initial assessment and triage process. The characteristics and clinical presentations of the first wave of patients are detailed in Table 1.

**Table 1:** Characteristics of the first wave of patients (n = 5). All five patients presented with minor injuries and were treated and discharged after basic medical care, except for two who required suturing for lacerations.

Nationality	Gender	Age (years)	Chief complaints	Triage	Outcome
Nepalese	Female	43	Contusions (arm and thigh)	Green	Discharged
Nepalese	Female	45	Oral laceration	Green	Sutured and discharged
Nepalese	Female	29	Headache and knee contusion	Green	Discharged
Nepalese	Male	33	Lip abrasion and tooth mobility	Green	Discharged
Nepalese	Female	28	Lower leg laceration	Green	Sutured and discharged

These five patients presented with relatively uniform injury patterns, facilitating efficient initial assessment and treatment. Notably, this group included cases requiring triage category modification based on evolving clinical findings.

Examination of injury patterns revealed a consistent trauma distribution reflective of the accident mechanism. Anterior-oriented injuries predominated, with facial trauma observed in eight patients and lower extremity injuries documented in 12 cases. Oral and dental trauma occurred in four patients, correlating with the impact dynamics of the collision.

The second wave of patients, arriving at 8:10 AM, presented greater clinical complexity, as shown in Table 2.

**Table 2:** Characteristics of the second wave of patients (n = 12). The second wave showed greater variability in injuries and required more intensive care. Two patients were upgraded from green to yellow during secondary triage due to the severity of their conditions.

Nationality	Gender	Age (years)	Chief complaints	Triage	Outcome
Nepalese	Female	22	Right radial styloid process fracture	Green	Splinted and discharged
Filipino	Female	27	Contusion of the right knee	Green	Discharged
Nepalese	Male	27	Contusions of the left lower leg and anterior chest	Green	Discharged
Nepalese	Female	23	Contusions of the face and left dorsum of the hand	Green	Discharged
Filipino	Female	22	Contusions of the forehead, left clavicle, right knee, and right ankle	Green	Discharged
Nepalese	Male	24	Contusions of both knees	Green	Discharged
Nepalese	Female	26	Contusion of the forehead, neck pain, and right shoulder pain	Green	Discharged
Nepalese	Male	30	Anterior chest and facial contusions	Green	Discharged
Japanese	Male	51	Abrasions of the forehead and both lower limbs	Green	Achieved hemostasis and discharged
Nepalese	Female	26	Contusions of the face, epistaxis, laceration of the lower lip, and contusions of both knees	Green	Discharged
Nepalese	Female	54	Contusion of the left mandible, injury to mandibular teeth	Green → Yellow	Referred to a specialist
Japanese	Male	64	Contusion of the right chest, fever (39.8 °C), Influenza A+	Green → Yellow	Treated and discharged

Notably, this group included cases requiring triage category modification based on evolving clinical findings. For this wave, additional staff were mobilized to enhance capacity. The response team for the second wave included twenty doctors, fifteen nurses, and ten administrative staff. This increased workforce allowed for comprehensive care delivery despite the higher clinical complexity of this group. Examination of injury patterns revealed a consistent trauma distribution reflective of the accident mechanism. Anterior-oriented injuries predominated, with facial trauma observed in eight patients and lower extremity injuries documented in twelve cases. Oral and dental trauma occurred in four patients, correlating with the impact dynamics of the collision.

Emergency response timeline

**Figure 3:**
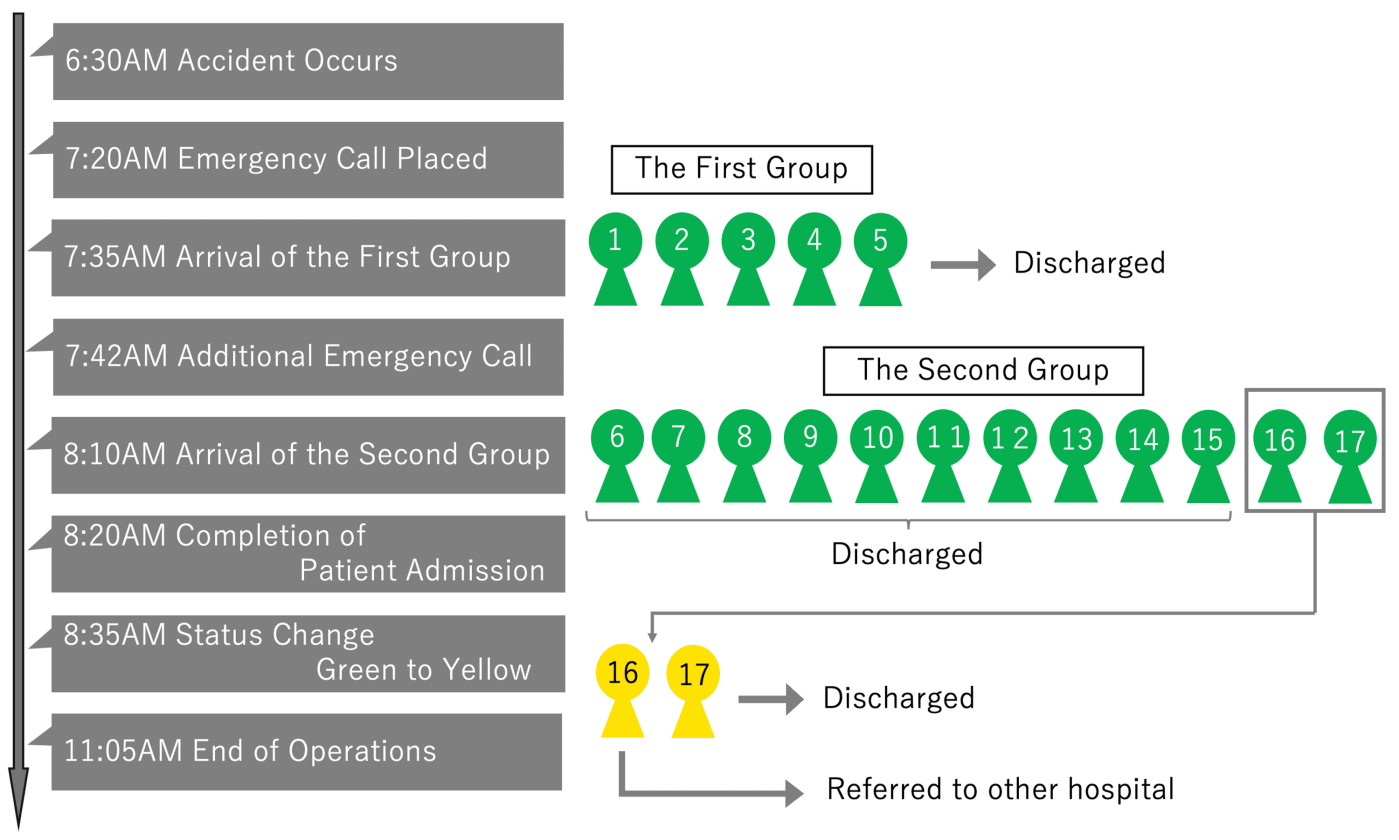
Timeline of the emergency response. This timeline outlines the key events of the emergency response following the mass casualty incident. The timeline includes the occurrence of the incident (6:30 AM), the initial emergency call (7:20 AM), the arrival of the first patient wave (7:35 AM), the arrival of the second wave (8:10 AM), and the completion of triage and operations (11:05 AM). Changes in triage status (green to yellow) and key interventions are also highlighted.

Figure 3 presents the chronological progression of the emergency response, highlighting key decision points and interventions.

During the initial triage, administrative staff recorded patient information, including ID, name, and date of birth, into the electronic medical record system (Fujitsu, Tokyo, Japan). Nurses and resident physicians conducted patient interviews, while orthopedic surgeons, general surgeons, emergency physicians, and internists collaborated to examine patients and prioritize diagnostics and treatments for those with higher severity.

The timeline also demonstrates the dynamic nature of the response, including triage category modifications and the implementation of infection control measures. Patient flow was managed using a large whiteboard in the emergency department, with updates recorded in real-time using markers for tracking.

Communication process and assessment dynamics

Implementation of the multi-modal communication strategy revealed notable differences in assessment durations between foreign and Japanese nationals. Assessment times for foreign patients averaged 22.3 minutes, compared to approximately 12.5 minutes for Japanese patients. This difference was attributed to several factors, including the need for name transcription into katakana, additional documentation requirements for foreign nationals, and complexities in multi-language medical communication. To address these challenges, staff used POCKETALK, a Japanese AI-powered translation device (Sourcenext Corporation, Tokyo). Emergency department nurses and administrative staff had received prior training on using the translation device and multilingual communication aids, ensuring efficient operation during the response.

Clinical management and outcomes

Patient management strategies were tailored to injury patterns and clinical presentations. The majority of patients (*n *= 15) received treatment and achieved discharge criteria during the initial assessment period. Two patients required specialist referral, including one case of mandibular trauma.

The detection of influenza A in one patient necessitated the implementation of isolation protocols, demonstrating the facility's capacity to manage concurrent medical conditions during a mass casualty response. Data were recorded in the electronic medical record system (Fujitsu) and later analyzed for insights into patient flow and resource utilization.

Implementation of systematic disaster response training

The experiences and challenges encountered during this MCI informed the development of a comprehensive disaster response training program. On February 17, 2024, our facility conducted an extensive disaster drill specifically designed to address the complexities of managing multilingual mass casualty scenarios. The training exercise incorporated both technical and cultural aspects of emergency response, with particular emphasis on communication challenges identified during the actual incident.

The drill scenario simulated a mass casualty event involving twenty mock patients, deliberately mirroring the demographic composition of the January incident. Initial triage was conducted using the START method, followed by secondary assessment using the PAT method, allowing participants to practice both protocols under time-constrained conditions. Simulated patients were recruited from local international communities and instructed to present with varying levels of English and Japanese language proficiency, creating realistic communication challenges.

The exercise implemented a structured communication protocol integrating electronic translation devices, multilingual medical cards, and pictorial communication tools. Medical staff practiced rapid patient registration techniques while maintaining accurate documentation in multiple languages. The simulation particularly focused on the critical first 30 minutes of mass casualty response, during which accurate triage and effective communication are paramount.

Comprehensive patient flow management protocols were tested, including the establishment of separate treatment zones based on triage categories and the implementation of a color-coded routing system to facilitate patient movement between departments. The exercise incorporated dynamic scenario progression, requiring participants to adapt to evolving patient conditions and modify triage categories accordingly. This approach specifically addressed the observation from the actual incident where two patients required triage category modification.

The real-time evaluation was conducted by experienced emergency medicine specialists who documented team performance metrics and communication efficiency. The exercise revealed several areas for protocol refinement, particularly in the initial registration process for foreign patients and the coordination of interpreter services. Observer feedback highlighted the need for expanded cultural competency training and standardization of multilingual documentation procedures.

**Figure 4:**
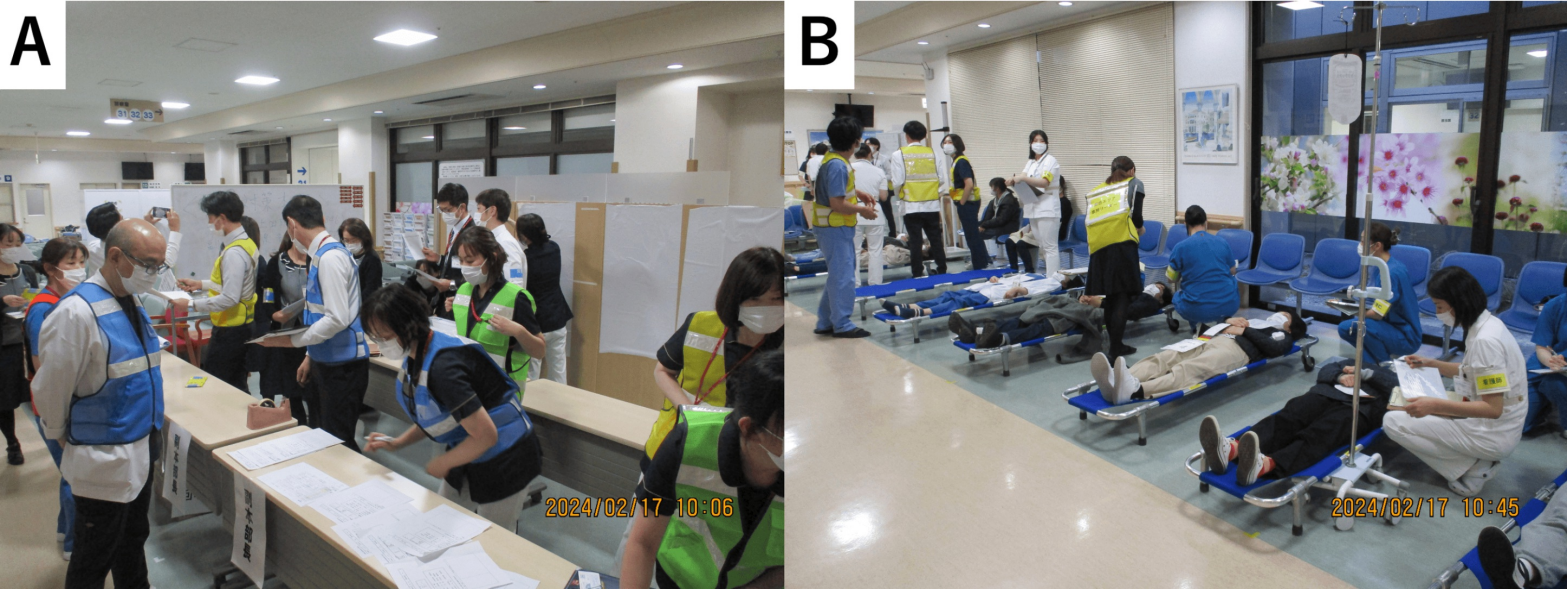
Mass casualty incident response training. This figure depicts the disaster drill conducted on February 17, 2024, at Fukuoka Seisyukai Hospital. Panel A shows the setup of the triage station and patient registration area, demonstrating the systematic organization for efficient patient categorization. Panel B illustrates the treatment area organization and patient flow simulation, emphasizing the hospital’s preparedness for handling mass casualty incidents. Individuals, including the hospital director and staff, appear in the images with their official consent for use in this publication.

Figure 4 illustrates key components of the training exercise, with Panel A showing the systematic organization of the triage station and patient registration area and Panel B demonstrating the treatment area configuration and patient flow patterns.

The physical layout was designed to optimize communication between different treatment zones while maintaining infection control standards.

## Discussion

The management of this MCI involving predominantly foreign nationals highlights several critical aspects of emergency medical response in an increasingly globalized healthcare environment. Our experience provides valuable insights into the challenges and opportunities for improvement in disaster medicine protocols, particularly in regions experiencing demographic transitions [1, 2].

The dynamic nature of triage decisions in this incident merits particular attention. The reclassification of two patients from green to yellow category demonstrates the limitations of initial START triage in scenarios involving language barriers [5]. The first case involved a female patient with mandibular trauma who, despite being ambulatory, was later found to have injuries requiring specialist intervention. The second case, involving the bus driver, illustrated how concurrent medical conditions can complicate trauma assessment. These observations align with Jenkins et al.'s findings regarding the significance of continuous reassessment in mass casualty triage, particularly when communication barriers exist [7]. The complexity of emergency triage in multilingual settings has been well documented by Pun et al.’s paper, which emphasizes the need for structured assessment protocols [8].

The influence of sociocultural factors on emergency medical care emerged as a significant theme in our analysis. The observed 9.8-minute difference in assessment times between foreign and Japanese patients reflects the complex interplay between language barriers and clinical evaluation. This finding corresponds with Flores' systematic review, which demonstrated how language barriers can significantly impact healthcare delivery timing and quality [3]. Language barriers and resource utilization studies have shown extended emergency department stays and increased resource requirements for patients with limited language proficiency [9,10].

The successful implementation of electronic translation devices and multilingual medical cards represents a practical application of Diamond et al.'s recommendations for culturally appropriate emergency services [11]. This approach aligns with research on interpreter use in pediatric emergency departments [12], which emphasizes the importance of structured communication protocols in multilingual emergency settings. Studies of medical interpreters in triage during mass-gathering incidents have demonstrated the vital role of language support systems in emergency care [10].

However, simultaneous disasters pose unique challenges, particularly in ensuring effective linguistic support for injured foreign nationals. Real-time communication may be hindered by the limited availability of translation devices or trained interpreters during large-scale emergencies. Future research should focus on scalable solutions, such as integrating AI-driven translation systems with centralized linguistic support hubs, to address these challenges. Additionally, improving cultural competency training for emergency staff could enhance their ability to listen actively and respond empathetically to foreign patients’ needs, even in high-pressure scenarios.

Our experience with distributed treatment areas revealed both advantages and challenges in mass casualty management. This dual-zone approach required additional coordination efforts, particularly for non-Japanese-speaking patients [11,13]. Recent studies on emergency department length of stay and utilization associated with primary language have highlighted the importance of efficient patient flow management [14]. The integration of infection control measures with trauma care protocols demonstrated our facility's ability to manage complex medical scenarios while maintaining standard precautions, as recommended by Hick et al. [15].

The incident highlighted the importance of regional emergency medical coordination, particularly for facilities serving diverse populations. Research has shown that perceptions of prehospital care for patients with limited language proficiency significantly influence emergency medical service delivery [16,17]. Our findings support Karliner et al.'s research showing that professional interpreters can improve clinical care efficiency [12]. The successful integration of various communication strategies reflects contemporary best practices in emergency medicine.

The subsequent implementation of a comprehensive disaster drill, incorporating lessons learned from this incident, represents a crucial step toward systematic improvement. The drill's focus on multilingual scenarios and cultural competency aligns with Idrose et al.'s recommendations for simulation-based disaster response training [18]. This approach to preparedness reflects an evolution in emergency medicine practice, acknowledging the growing importance of cultural and linguistic competency in healthcare delivery.

Our study has several limitations as a single-center experience, with findings that may not be fully generalizable to facilities with different demographic profiles or resource availability. Also, our study's findings are based on a relatively homogeneous group of foreign nationals, with the majority being from Nepal. This limitation reflects the specific demographic characteristics of the incident and the local population distribution. Consequently, the results may not fully capture the challenges and needs associated with more diverse foreign populations. The relatively small sample size limits statistical analysis of outcome measures, and the specific cultural and linguistic background of our patient population may not represent the full spectrum of challenges faced by other institutions. Future studies should aim to include a broader range of nationalities to improve generalizability and address varying cultural and linguistic needs.

The longer assessment times for foreign patients highlighted in this study underscore the need for enhanced multilingual support systems. Specifically, improving the efficiency of translation tools, streamlining documentation processes for foreign nationals, and standardizing multilingual triage protocols are critical steps. Future disaster drills should incorporate scenarios reflecting these challenges to train staff in handling such complexities under time constraints.

Looking to the future, our experience suggests the necessity of enhancing translation services and multilingual medical documentation, developing cultural competency training programs, and modifying triage protocols. These recommendations align closely with the observed challenges and are intended to provide actionable strategies for hospitals facing similar scenarios. The establishment of integrated regional emergency medical networks specifically equipped to handle MCIs involving foreign nationals will become increasingly crucial as Japan's demographic landscape continues to evolve.

To support broader applicability, we recommend that other hospitals consider tailoring these strategies to their unique demographic and resource contexts. For example, institutions in areas with high concentrations of foreign workers could prioritize recruiting multilingual staff, establishing centralized linguistic support hubs, or investing in AI-driven translation devices. These measures can help bridge the gap between current capabilities and the growing demand for culturally and linguistically competent emergency medical care.

These developments must occur within the context of existing emergency medical systems while maintaining the highest standards of patient care and safety [19].

These insights and recommendations from our experience have particular relevance given Japan's growing foreign worker population and the increasing likelihood of similar incidents in the future. The successful management of this case, despite identified challenges, provides a valuable template for other secondary emergency facilities adapting to demographic changes in contemporary Japanese society.

## Conclusions

This MCI predominantly involving foreign workers revealed several critical insights. The importance of dynamic triage was demonstrated through the reclassification of two patients, highlighting the necessity for continuous reassessment, particularly in scenarios where language barriers exist. Although electronic translation devices proved helpful in overcoming communication challenges, the prolonged assessment times for foreign patients underscored the need for more efficient multilingual support systems. Additionally, the experience emphasized the value of structured protocols tailored to managing multicultural patient groups in emergency settings.

Based on these findings, we recommend the implementation of standardized multilingual triage protocols and the establishment of dedicated language support systems within emergency departments. Furthermore, regular disaster drills incorporating scenarios involving foreign patients should be conducted to better prepare for such incidents. As Japan's foreign worker population continues to grow, these adaptations will be essential for ensuring effective and equitable emergency medical care.
